# Multimodality management and outcomes of brain arterio-venous malformations (AVMs) in children: personal experience and review of the literature, with specific emphasis on age at first AVM bleed

**DOI:** 10.1007/s00381-017-3383-4

**Published:** 2017-03-21

**Authors:** Anan Shtaya, John Millar, Owen Sparrow

**Affiliations:** 1 Wessex Neurological Centre, University Hospital Southampton, Southampton, UK; 2 Atkinson Morley Neurosurgery Centre, Academic Neurosurgery Unit, St George’s, University of London, London, SW17 0RE UK

**Keywords:** Ruptured AVMs, DSA, Microsurgery, Embolisation, Radiosurgery, Outcome

## Abstract

**Purpose:**

The purpose of this paper is to study the presentation and analyse the results of multimodality treatment of brain arterio-venous malformations (AVMs) in children at our centre and review age at first AVM rupture in the literature.

**Methods:**

Of 52 patients aged <18 years, 47 with brain AVMs (27 males and 20 females) aged 4–17 years (mean 12.2) were retrospectively reviewed. PubMed search revealed five additional studies including 267 patients where the prevalence of age-related AVMs rupture was analysed.

**Results:**

In our study, 37 patients had bled, 9 were symptomatic without haemorrhage and 1 was incidental. Spetzler-Martin score distribution was 5 cases grade I, 18 grade II, 21 grade III and 3 grade IV. Appropriate imaging was performed, either CT/MRI angiogram only (in emergency cases) or catheter angiogram, prior to definitive treatment. There were 40 supratentorial and 7 infratentorial AVMs. Twenty-nine patients had microsurgery alone and 9 patients were treated by radiosurgery only. Three patients were embolised, all followed by radiosurgery, with one requiring surgery too, while 4 patients had combined surgery and radiosurgery. One patient is awaiting radiosurgery while another was not treated. Good outcomes, classified as modified Rankin score (mRS) 0–2 improved significantly after intervention to 89.4% from 38.3% pre-treatment (*p* value <0.0001). Angiography confirmed 96.6% obliteration after first planned operation. Repeat cerebral angiogram around age 18 was negative in all previously cured patients. Reviewing the literature, 82.0% (95% CI = [77–87]; *N* = 267) of children diagnosed with brain AVMs (mean age 11.4 ± 0.4) presented with a bleed in the last 22 years. Males significantly outnumbered females (136 vs 84) (*p* < 0.001). Ninety-five patients underwent surgical intervention alone when compared to other treatment modalities (*p* < 0.001).

**Conclusions:**

Microsurgical excision of surgically accessible intracranial AVMs remains the primary treatment option with very good outcomes. A significant number of patients’ AVMs ruptured around puberty; therefore, understanding the pathophysiology of AVM instability at this age may aid future therapy.

## Introduction

Impaired embryological differentiation of primordial vascular channels is believed to have a significant congenital role in developing brain arterio-venous malformation (AVM) anomalies [1, 2]. About 12–18% of patients who presented with brain AVMs are among children [3]. Brain AVMs result in 35%–55% of haemorrhagic strokes in children, with an incidence of 1.4 per 100,000 person-years and significant mortalities and morbidities [4, 5]. In one series, mortality reached 25% when averaged over 40 years, with an improvement from 39 to 16% over this time, coinciding with technological advances [6]. The annual bleeding risk of ruptured AVMs in children was reported as 2.71% and increased when associated with aneurysms and deep venous drainage [7]. In the Blauwblomme et al. study, AVMs ruptured at a mean age of 9.7 years (range 7.0–12.7). Furthermore, assuming that AVMs are congenital lesions, they estimated that the annual bleeding risk is 6.3% with significant associations with female sex (49.4% females vs 37.5% males) and annual re-haemorrhage rate of 14.8% [8]. The number of patients that have been diagnosed with AVMs in the neonatal period or infancy is very small [9, 10] and none to date has been diagnosed in utero in spite of the advances in radiological diagnostic techniques, including the now routine use of good quality ultrasound imaging [11]. Another recent study reported a mean age of AVM rupture of 11.3 years [12]. Thus, the vast majority of children with ruptured AVMs presented around or after the onset of puberty. This observation raises questions about the pathophysiology of AVMs and whether pubertal changes induce instability.

AVM treatment is essentially aiming at achieving angiographic cure (abolition of arterio-venous shunting) with no or minimal risk. This can be achieved by either surgical excision of the AVM nidus, endovascular complete AVM nidus embolisation, radiosurgical thrombo-obliteration or by a combination of these modalities. Previous studies have shown that up to 18% of children with AVMs were deemed untreatable [10, 13]. This may well be down in part to difficulties in applying multidisciplinary methods when addressing such complex cases. However, flexibility, expertise and approaching AVMs with more than one modality of treatment available improve the outcomes and reduce morbidity and mortality [7, 12].

In our centre, a multidisciplinary team of neurovascular neurosurgeons, interventional neuroradiologists and input as appropriate from our radiosurgery referral centre were utilised to plan treatment for children with AVMs over the last 22 years. The most suitable individual treatment strategy was developed, consisting of surgical AVM resection, endovascular embolisation, radiosurgery or a combined treatment policy.

The purpose of this study is to present our experience since 1994 using this multimodality treatment approach in the management of ruptured and unruptured paediatric brain AVMs and review age-related AVMs rupture risk in the literature.

## Methods

A prospectively maintained database (OCS-senior author) of vascular malformation patients treated at Wessex Neurological Centre was searched to identify all patients whose first AVM presentation/treatment occurred before 18 years of age between 1994 and 2016. We initially identified 52 patients. Of these, 47 individuals (27 males and 20 females) were confirmed to have brain AVMs, so were included in the retrospective analysis. The five excluded patients were a single AVM patient—seen after the 18th birthday, one with a traumatic carotico-cavernous fistula, two patients with scalp fistulae (so-called “cirsoid aneurysms”), and the last one had a simple *sinus pericranii*. AVMs were primarily diagnosed and followed up using catheter cerebral digital subtraction angiography (DSA). Occasionally, in cases of acutely ruptured AVMs with significant mass effect, computed tomographic angiography (CTA) or magnetic resonance angiography (MRA) was considered adequate to delineate the AVM nidus and angio-architecture. All the resected AVMs were also verified by histopathological examination. All cases have been discussed by the neurovascular multidisciplinary team, which includes other vascular neurosurgeons and interventional neuroradiologists. AVMs were classified according to the Spetzler-Martin grading system, and location was noted. Retrospective outcome analysis using a dichotomised Modified Rankin Scale score (mRS 0–2 good outcome, 3–6 poor outcome) was performed after stabilising the patient and/or seizure control before surgery, and at follow-up. Any intraoperative or postoperative complications were analysed. A PubMed search was conducted for ruptured brain AVMs in children. We scanned titles, abstracts and whole papers of relevant information. Five studies included data about age/sex when AVMs ruptured, so were eligible for inclusion in our review. Two hundred and sixty-seven patients were included (five literature studies and our series) of whom 220 patients presented with AVM rupture. Studies lacking the relevant information specific to our review were not included. The collected data were analysed retrospectively using Microsoft Office Excel 2013 (Microsoft, USA). Statistical analysis using Graph Pad Prism (USA) Student’s *t* test or the chi-square test was performed as appropriate. Period prevalence for ruptured AVMs with 95% confidence intervals (CI) was calculated using the Clopper-Pearson exact test. *P* value <0.05 was considered significant.

## Results

**Fig. 1:**
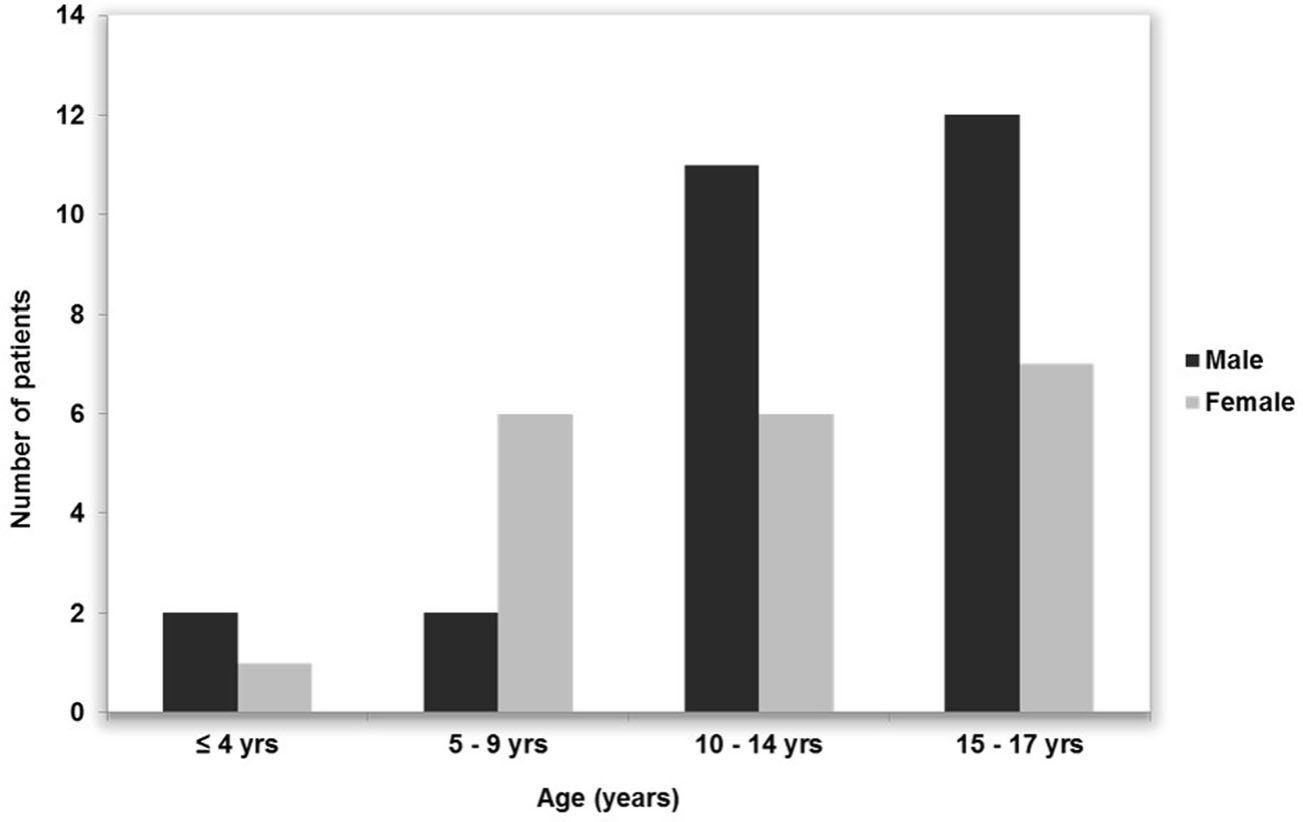
All patients’ demographics. Age and sex distribution at diagnosis

Figure 1 illustrates patient demographics. There were 27 males and 20 females with a mean age of 12.2 years (range, 4–17 years). Of these 47 patients, 37 (78.7%) had bled. Seventy-nine percent (95% CI = [64–89]; *N* = 47) of children who were diagnosed with AVMs (mean age 12.1 ± 0.6) presented with rupture over the last 22 years. Patients who presented with a bleed had either headache, reduced level of consciousness or both. Nine (19.2%) patients were symptomatic without haemorrhage, of whom five had seizures and four were only complaining of headaches. In one patient (2.1%), an AVM was diagnosed incidentally.

On admission to the Wessex Neurological Centre 18 patients had a Glasgow Coma Score (GCS) of 15, 10 patients were GCS 13–14, 2 patients had GCS 9–12, and 16 patients were GCS of ≤8. One patient was initially treated at another centre; hence, the GCS is not recorded.

### AVM location

**Fig. 2:**
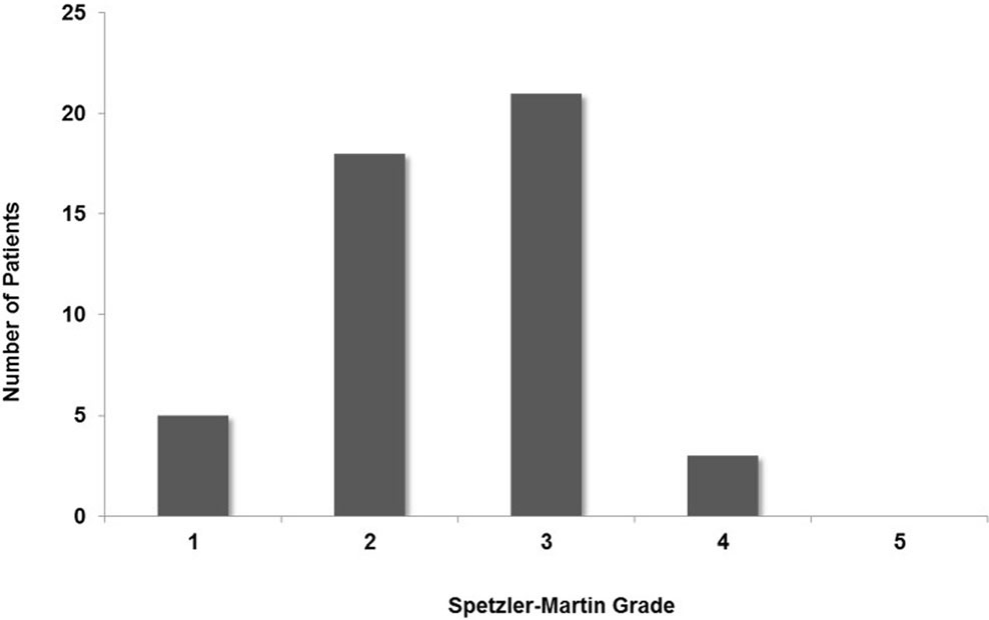
AVM grading. Patients’ AVM classification according to Spetzler and Martin AVM grading

In 40 patients, the AVM was supratentorial (frontal lobe 9, parietal lobe 7, temporal lobe 7, occipital lobe 6, parieto-occipital 3, temporo-parietal 2, fronto-temporal 1, basal ganglia and/or thalamus 3, corpus callosum 2) and infratentorial in the other 7 (all cerebellar) (Table 1). The Spetzler-Martin grade (Fig. 2) was as follows: grade 1 in 5 cases, grade 2 in 18, grade 3 in 21, and 3 were grade 4. In 3 patients (6.4%), the AVM was associated with a flow-related aneurysm.

**Table 1:** AVM localisation. This table demonstrates the numbers and location of the AVMs

Location	Number of patients (%)
Lobar location	35 (74.4%)
Frontal	9 (19.1%)
Pariental	7 (14.9%)
Temporal	7 (14.9%)
Occipital	6 (12.8%)
Fronto-temporal	1 (2.1%)
Temporo-pariental	2 (4.3%)
Parieto-occipital	3 (6.4%)
Thalamus/basal ganglia	3 (6.4%)
Corpus callosum	2 (4.3%)
Cerebellum	7 (14.9%)

Patients underwent digital subtraction angiography (DSA) and MRI with MR angiography (MRA) when possible, were discussed in the neurovascular meeting as appropriate and the most suitable modality of treatment was arranged. Twenty-nine patients had microsurgery alone; while in nine patients, radiosurgery only was sufficient to obliterate the AVM. One further recently diagnosed patient is awaiting radiosurgery. Three patients were embolised, all followed by radiosurgery, with one requiring microsurgery too. Four patients required a combined approach with surgery and radiosurgery to achieve satisfactory results, see Table 2. One patient arrived moribund with failing circulation despite huge doses of inotropes, in coma (GCS 3) with unreactive pupils unresponsive to mannitol and was not treated.

**Table 2:** Treatment modalities. We show the different treatment options our patient underwent and their percentages among all

Method	Number of patients (%)
Surgery (only)	29 (61.7)
Total excision first definitive procedure	28 (59.6%)
Total excision second definitive procedure	1 (2.1%)
Haematoma evacuation as first procedure	5 (10.6%)
CSF Diversion (temporary)	7 (14.9%)
CSF Diversion (permanent-shunt)	2 (4.3%)
Radiosurgery (only)	10 (21.3)^a^
Endovascular embolisation and radiosurgery	3 (7%)
Embolisation, radiosurgery and surgery	1 (2.1%)
Surgery and radiosurgery	4 (8.5%)
No treatment	1 (2.1%)^b^

^a^One patient is awaiting radiosurgery

^b^One patient arrived moribund, unresponsive to mannitol, therefore no further treatment

In 83% (39/47) of the patients described, an angiographic cure of the AVM was achieved after treatment. One recently diagnosed patient (2.1%) is awaiting radiosurgery treatment. Six patients (12.8%) had residual AVM after the planned treatment had been completed. One patient was from the surgical group, 2 patients were from the radiosurgery group and three patients were from those who were managed with more than one planned modality of treatment (but when all had been completed). Patients who were suitable candidates for radiosurgery were referred to the Sheffield Radiosurgery Centre, where they underwent that treatment only. However, we have followed all up. An experienced interventional neuroradiologist (JM) at the Wessex Neurological Centre performed endovascular treatments.

Evacuation of a space-occupying haematoma was necessary in five patients among the 37 with ruptured AVMs (10.6%). In one patient, the haematoma was acutely evacuated with simultaneous surgical resection of the AVM. In the remaining four patients, the haematoma was evacuated without removal of the AVM. Seven patients required insertion of an external ventricular drain and subsequently two patients required permanent ventriculo-peritoneal shunts to divert CSF (Table 2).

Surgery was performed following the standard principles of microsurgical AVM resection. Image guidance was used when available and deemed advantageous. AVM localisation and identification was sometimes difficult, when small or somewhat diffuse, planning being conducted using a combination of DSA, CTA and MRI/MRA employing image guidance.

**Fig. 3:**
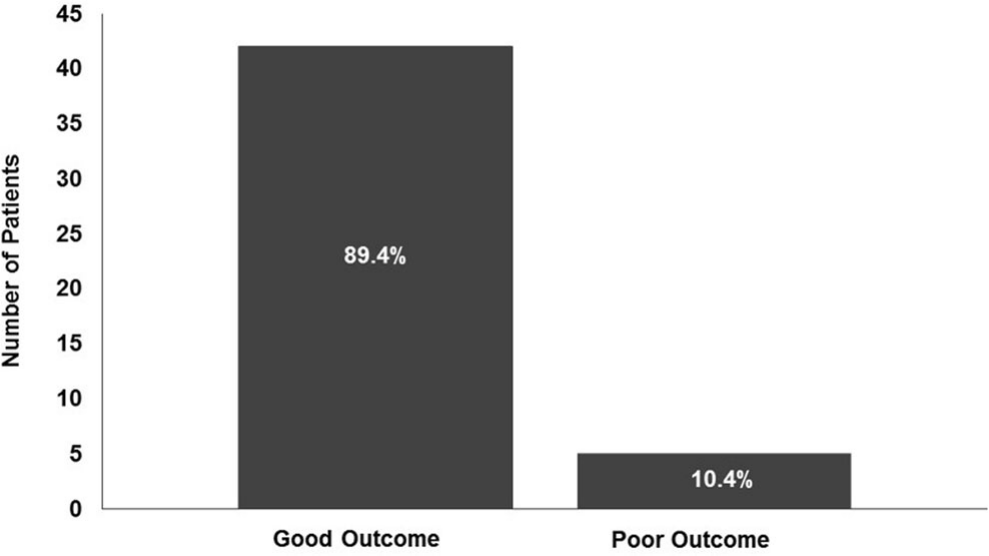
mRS scores. This figure demonstrates good outcome (mRS 0–2) compared to poor outcome (mRS 3–6) on follow-up after treatment was completed. We report significant improvement in good outcome

Clinical outcomes were measured by mRS and compared initial mRS at first presentation, after stabilising the patient and/or seizure control, to that either at discharge or at the latest follow-up clinic visit. The results are displayed in Fig. 3. Good outcomes, classified as mRS 0–2, improved significantly after intervention to 89.4% from 38.3% pre-treatment (*p* value <0.0001). The breakdown was mRS 0 in 4 patients pre-surgery and 16 after treatment, mRS 1 in 5 cases pre-surgery and 14 on follow-up, mRS 2 in 9 patients pre-operation and in 12 on last clinic visit, mRS 3 in 4 cases pre-surgery and 3 on follow-up, mRS 4 in 9 patients pre-surgery but only 1 on follow-up, mRS 5 in 16 patients pre-surgery and none on last clinic visit, and finally one patient who was mRS 5 on admission was not treated and succumbed (mRS 6).

Imaging confirmed obliteration of AVMs in 29 cases from the surgery only group, while 7 patients who had radiosurgery only were cured. One patient is awaiting radiosurgery treatment. The rest underwent more than one treatment modality before achieving cure. We noted that para-trigonal AVMs with intraventricular haemorrhage were difficult to treat, often necessitating more than one modality of treatment. Some patients required more than one operation and/or more than one session of radiosurgery. Catheter cerebral angiography around age 18 is performed in all paediatric AVM patients, once vascular plasticity is assumed to have reduced. It has proved reassuringly negative in all patients who had been deemed cured previously. Of the nine patients who initially had seizures with/without haematoma, seven were seizure-free at last clinical follow-up. However, one patient developed seizures a few months after surgery and another one developed seizures 1 year after radiosurgery.

We observed transient procedure-related morbidity in three patients who had undergone emergency operations to either evacuate haematomas or divert CSF to reduce intracranial pressure. Of these, one patient developed ventriculitis while another had a CSF infection. Both were treated successfully with good outcome. A third patient, who developed wound infection, required antibiotics. Over the longer term, one patient developed a subdural hygroma and two additional patients needed permanent ventriculo-peritoneal shunts after acute hydrocephalus had required EVD insertion.

## Discussion

Although the prevalence of childhood AVMs is difficult to assess, it has been suggested that it is less than 1% at autopsy; however, this may have changed recently with the frequent use of modern imaging modalities leading to more incidental AVMs being diagnosed [14]. The annual risk of AVM haemorrhage in children is approximately 2–6% per annum (assuming presence from birth) and is higher in the first year post rupture [7, 8, 15–18]. However, age-related prevalence of haemorrhage among children diagnosed with brain AVMs is not clear in the literature. In spite of the proposed congenital theory of AVMs formation, the number of neonates and young children diagnosed/treated for such pathology is very small [1, 7, 8, 17]. Furthermore, recurrence of brain AVMs after angiographic cure has been reported in a number of cases [19–22]. Interestingly, Yeo et al. reported diagnosing de novo brain AVMs in two children where MRI scans (as a workup for seizures) were normal years earlier [23]. Altogether, this may suggest multifactorial pathophysiology of brain AVMs and/or their instability rather than a purely congenital origin and an even subsequent annual risk of rupture.

Brain AVMs in the paediatric age group account for 30–50% of haemorrhagic strokes [3, 24]. A high percentage of children with AVMs, often more than 60%, present with rupture [7, 8, 15, 17, 25]. In our series, 78.7% presented with haemorrhage. It has also been reported that children often present with haemorrhage from AVMs when compared to their adult counterparts [26]. Indeed, some published series report haemorrhage rates of 80–85%, resulting in mortality of up to 25% [6, 13, 25]. Importantly, the natural history of untreated ruptured brain AVMs in children is not favourable, with high morbidity and mortality [27]. Over one third of patients present with or develop seizures due to brain AVMs and/or haemorrhage [11, 28]; in our study, 19% of patients had seizures at presentation but few of them remained on antiepileptic medication on last follow-up. However, a further 4.2% developed seizures months to years after treatment. Overall, we report 9% long-term seizures in our series. Given the reportedly high morbidity and mortality of brain AVMs in children, it is therefore crucial to outline a strategy to implement the optimum treatment modality to achieve permanent obliteration of the AVM nidus with minimal risks. Although the previously published series of AVMs in children came from well-known large neurosurgery centres, the number of patients in each study is small. We report 47 patients of whom 33 had surgery; mostly (29) surgery only. We also demonstrate a very low mortality rate (2.1%) at our tertiary referral centre with a catchment population of around 3 million people over a period of more than 22 years.

**Fig. 4:**
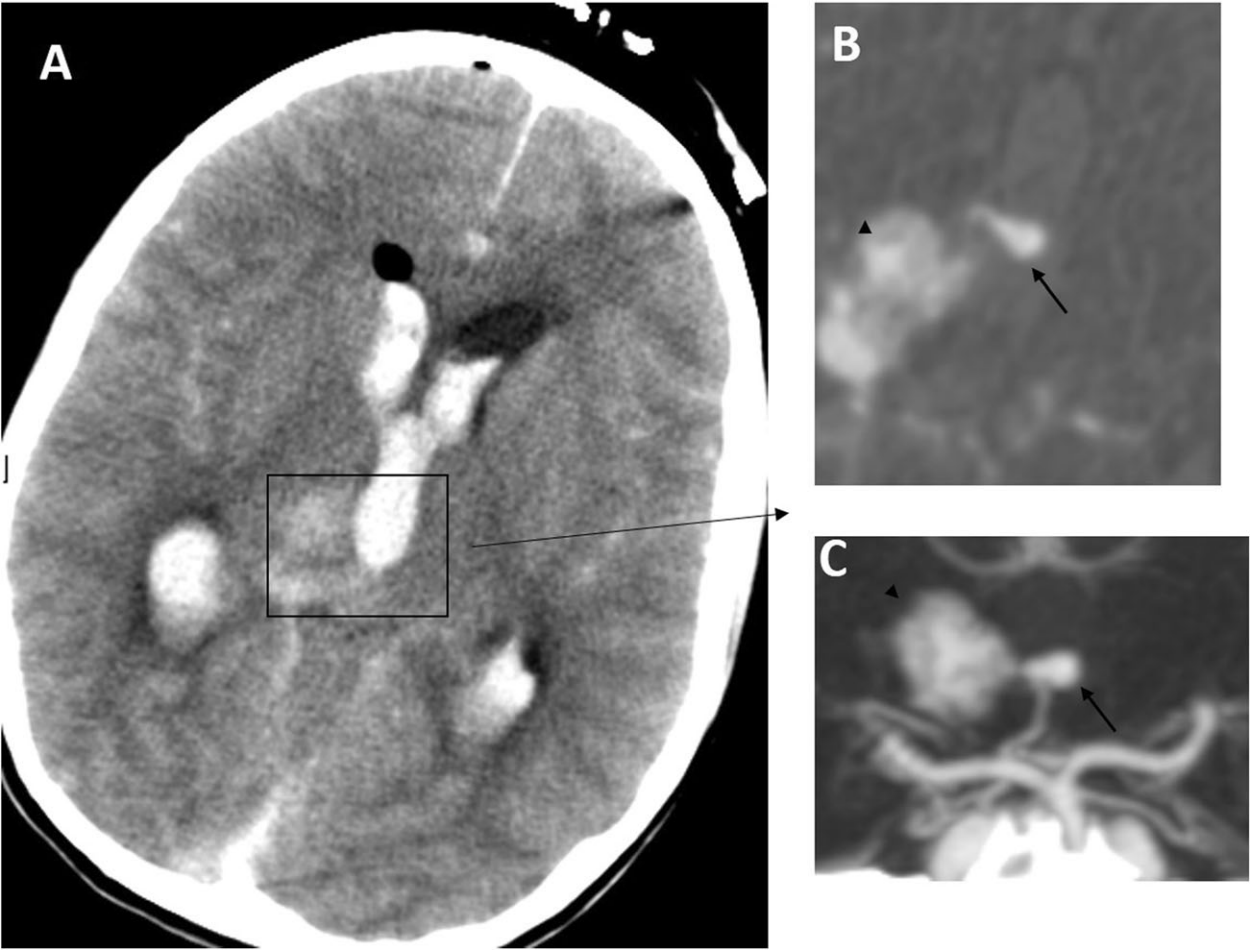
Case illustration. **a** Pre-treatment CT, which shows a right thalamic bleed with intraventricular extension and hydrocephalus after external drainage placement. **b** Axial CT angiogram image reveals the Percheron artery flow aneurysm projecting into the third ventricle (*arrow*) and AVM nidus (*arrowhead*). **c** A 3D CT angiogram picture that delineates the flow aneurysm (*arrow*) and the AVM nidus (*arrowhead*)

**Fig. 5:**
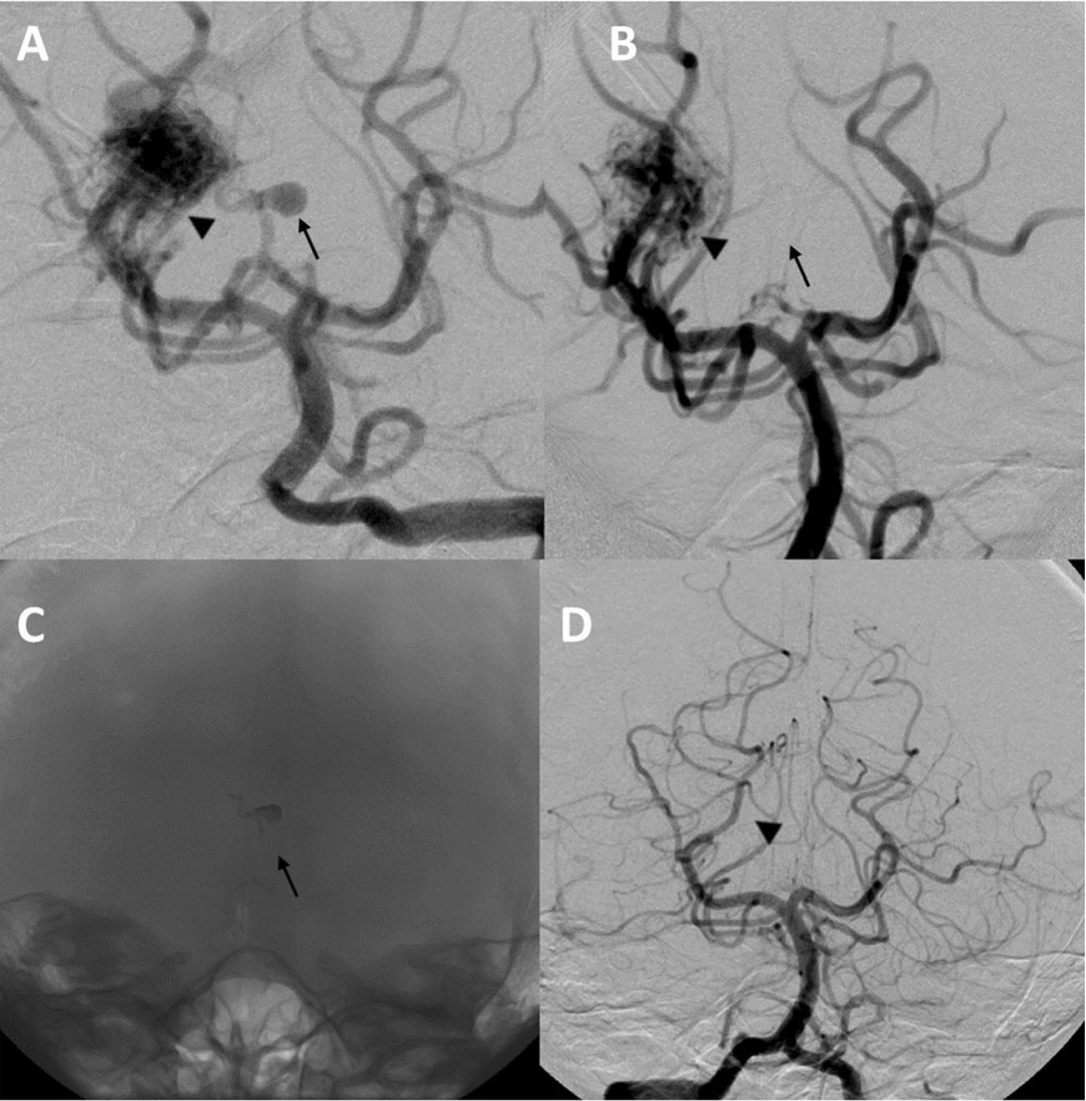
DSA images. **a** Pre-treatment DSA picture reveals the aneurysm (*arrow*) and the AVM nidus (*arrowhead*). **b** Post-embolisation DSA demonstrates occlusion of the aneurysm. Follow-up images demonstrating the “Onyx” cast (*arrow*) in the flow aneurysm (**c**) and a DSA reveals obliteration of the AVM (*arrowhead*) (**d**)

In common with others [7, 15], we advocate a multidisciplinary approach utilising multimodality treatment options including combining different treatment strategies to maximise the permanent nidus obliteration rate with low treatment morbidity. Our results and recent reports [7, 12, 15] add support to this concept. As an example, Fig. 4 demonstrates a right thalamic haemorrhage from an aneurysm on the artery of Percheron, in turn feeding the AVM. The patient presented with sudden onset headache and collapse with a GCS of 4/15; she had an emergency EVD followed by DSA and embolisation of the aneurysm and feeder using an ethylene vinyl alcohol copolymer (Onyx™) followed by radiosurgery with complete AVM/aneurysm obliteration (Fig. 5) and good recovery to mRS of 3. In centres where only a single or a combination of two treatment modalities is available, the number of patients in whom the treatment risks are deemed to exceed the calculated risks of natural history, and who therefore remain untreated, is comparatively high [13]. The availability of better neurosurgical/interventional facilities, including radiosurgery centres in the UK, has encouraged us and others to utilise more active and appropriate treatment strategies, yielding improved outcomes.

Multidisciplinary review and the use of a balanced multimodality approach in the management of paediatric brain AVMs reduce procedure-related morbidity and mortality, and increase treatment efficacy. In our study, we demonstrate significant improvement in outcome (mRS 0–2 in 89.4% after intervention, from 38.3% after initial resuscitation) (Fig. 3).

Microsurgery, plays a key role in every therapeutic strategy for the management of both adult and paediatric brain AVMs. However, surgery should remain one component in the armamentarium for AVM management and there is a risk that surgical approaches outside a balanced multimodality treatment strategy can result in higher procedural morbidity rates and poorer neurological outcomes when compared to combined treatment plans [25]. This applies particularly to high Spetzler-Martin grade lesions, such as the larger and deep AVMs [15]. Thirty-three cases underwent surgical intervention in our series, of which 29 patients had surgery alone. Looking at the ruptured AVMs group in our study, we noted that 25 patients had surgery only. The decision to recommend surgery depended on many factors including MDT discussion, Spetzler-Martin grade and the accessibility of the lesion with minimal risk. We noted that deep AVMs, eloquent and para-trigonal lesions were often difficult to obliterate surgically, requiring a multimodality approach.

In our study, emergency evacuation of haematomas was required in five patients only of the ruptured brain AVMs treated. Seven patients required EVD placement, of whom two required permanent V-P shunts (Table 2). The ruptured AVMs were mostly treated in a delayed fashion after DSA and MRI/MRA, and discussion at the MDT meeting. The treatment options included a combination of different treatment modalities taking account of potential procedural complications, including sequential risks of more than one treatment, balanced against the risk of AVM re-bleeding (Figs. 4 and 5). Since protection from AVM re-bleeding is provided only after angiographic documentation of permanent nidus and arterio-venous shunt obliteration, haematoma evacuation without surgical AVM removal as well as subtotal nidus resection are acceptable only as emergency measures within a treatment strategy aiming at definitive cure of the malformation on an elective or semi-elective basis. All cases undergo DSA after 3 months, to assess the efficacy of surgery. We describe 39 (83%) patients with negative cerebral catheter angiogram after initial treatment. Six patients had residual AVM at follow-up (1 from the surgical group only, 2 from the radiosurgery group and 3 from the combined treatment group) (Table1). Initial treatment was planned as surgery alone, radiosurgery only, embolisation alone or a planned combined approach. This combined approach was needed in 2 cases where patients had surgery then radiosurgery. This is considered a high success rate after the initial treatment. Moreover, repeat DSA at 18 years old for those who have reached that age has remained negative, and therefore these patients have been discharged.

The conventional model is that AVMs are congenital lesions that arise from the abnormal development of the arteriolo-capillary network between the arterial and venous circulations within an organ [29]. However, the timing, stimulus and early architectural deficiencies in the AVM are not well understood. It has long been thought that the defects occur before the embryo is 44-mm in length (this is the stage of definitive formation of the adult pattern of arterial wall structure) [29]. To date and in spite of the advances in antenatal diagnosis of foetal anomalies, we could identify no reports of brain parenchymal AVMs diagnosed in utero. This may suggest that either these lesions have not developed yet or else they have not reached a size where they have become visible with the techniques currently available. Literature reports suggest most children diagnosed with AVMs are in their adolescence, with a small number of patients under the age of 8 years, and hardly any under the age of 1 [1, 3, 10, 12, 14, 15, 17, 25]. In our series, the mean age was 12.2 ± 3.8, matching the age of adolescence. We had 20 female patients of whom only 7 were under the age of 10. The other 27 were males, only 4 being under the age of 10. It may be significant that puberty commences at a younger age in females than males in developed countries. There will inevitably be some patients who were treated urgently elsewhere; however, the vast majority will have been treated and followed up in our unit. The absence of infants or neonates is noteworthy, an observation common to other studies. We propose the mechanisms of AVM formation (and/or enlargement) or perhaps a period of instability commencing around the age of puberty, provokes the clustering of bleeds around this age, and warrants further consideration. As we noted in our study, in common with others, most of these patients present with a bleed during puberty/adolescence, casting some doubt on the mechanism of AVM formation being purely congenital. In addition, recurrence of brain AVMs after angiographic cure suggests a possible alternative mechanism of AVMs formation [20, 22]. In line with this theory, we have further reviewed the literature and identified, in addition to our series, five more studies [7, 12, 17, 25, 26] where age/sex at rupture of AVMs, where patients’ demographics were available for analysis. There were 267 patients included in the review (Table 3). Two hundred and twenty patients presented with rupture of their AVMs. In our literature review we report that 82% (95% CI = [77–87]; *N* = 267) of children diagnosed with brain AVMs (mean age 11.4 ± 0.4) presented with rupture over the last 22 years included. In spite of a previous report of increased risk of haemorrhage associated with female sex [8], our review revealed a significant difference in gender-related AVM rupture (136 males vs 84 females) (*p* < 0.001). These findings perhaps propose that hormonal changes around puberty may be related, and suggest that the sex difference may be attributable to the hormonal differences too. Unfortunately, we did not perform any sex hormone measurements in our cases. We further analysed the available data of modality of treatment for 201 patients (Table 3), which shows that 95 had surgery only, 23 underwent radiosurgery alone, 12 had embolisation alone and 71 had combined approaches. Surgical intervention was significantly higher than any other treatment modality with good outcomes (*p* < 0.001). Regarding AVM pathophysiology, various animal models have implicated sex hormones in playing an important role in angiogenesis through vascular endothelial growth factor (VEGF) and its receptors [30–32]. Inhibiting angiogenesis using doxycycline has even been proposed to have a role in the treatment of AVMs in patients [33]. Interestingly, it has been reported that testosterone remodels aortic wall in arterio-venous fistulae [34]. These studies may suggest that enhancement of angiogenesis along with other factors, currently obscure, may have an important role in generating instability in AVMs or even provoking AVM formation. Our review (Table 3) shows that approximately 62% of ruptured AVMs are among males and whether there is a co-relation with hormonal changes and/or testosterone levels is not known. Further research in this field may lead to novel therapy applicable even to the inoperable cases.

**Table 3:** Literature review studies. This table shows the six studies included in our review, the total number of patients who are below 18 years old, the number of patients and mean age at rupture, sex distribution and the modalities of treatment specifically for ruptured AVMs

Series	Total number of patients	Ruptured AVMs (%)	Mean age at rupture in years	Sex (ruptured only)		Treatment modality of ruptured AVMs (%)	
				M	F		
Nerva et al. 2016	40	27 (68)	11.3	17	10	Surgery alone	8 (30)
						Radiosurgery	6 (22)
						Embolisation alone	0
						Combined	13 (48)
Abecassis et al. 2016	22^a^	14 (64)	12.2	11	3	Surgery alone	3 (21)
						Radiosurgery alone	3 (21)
						Embolisation alone	0
						Combined	8 (58)
Blauwbiomme et al. 2014	106	106(100)^b^	9.7	62	44	Surgery alone	43 (40.6)
						Radiosurgery alone	8 (7.5)
						Embolisation alone	12 (11.3)
						Combined	43(40.6)
Sanchez-Mejia et al. 2006	32	19 (54.4)	11.3	13	6	Surgery alone	–
						Radiosurgery alone	–
						Embolisation alone	–
						Combined	–
Kiris et al. 2005	20	17(85)	12	12	5	Surgery alone	16(94)
						Radiosurgery alone	0
						Embolisation alone	0
						Combined	0
Shtaya et al. (current series)	47	37(79)	12.1	21	16	Surgery alone	25
						Radiosurgery alone	6^c^
						Embolisation alone	0
						Combined	6^d^

^a^Patients 18 and above were excluded

^b^This is a ruptured AVMs series only

^c^One patient is awaiting radiosurgery

^d^One patient did not have any treatment (died)

To conclude, although brain AVMs in children are rare, they represent a serious lifelong risk of haemorrhage and neurological deficits. Most children will present with an intracranial bleed when compared to the adult population; a multidisciplinary discussion and approach should to be standard for each case, considering the various treatment options available. Endovascular embolisation is usually utilised as an adjunct, combined with either surgery, radiosurgery or both to achieve better cure rates. Microsurgical excision is the treatment of choice in low-grade, accessible and non-eloquent AVMs with very good outcomes. Though a congenital aetiology has been assumed, as most patients present in adolescence, there must be some doubt over either their formation or alternative explanations for this clustering in age of haemorrhages. Further investigation of the pathophysiology and/or stability of AVMs at various ages may lead to new treatment strategies.
